# Identifying genes within pathways in unannotated genomes with PaGeSearch

**DOI:** 10.1101/gr.278566.123

**Published:** 2024-05

**Authors:** Sohyoung Won, Jaewoong Yu, Heebal Kim

**Affiliations:** 1 Interdisciplinary Program in Bioinformatics, Seoul National University, Seoul, Republic of Korea, 08826;; 2 eGnome, Incorporated, Seoul, Republic of Korea, 05836;; 3 UNGENE, Incorporated, Seoul, Republic of Korea, 14556;; 4 Department of Agricultural Biotechnology and Research Institute for Agriculture and Life Sciences, Seoul National University, Seoul, Republic of Korea, 08826

## Abstract

In biological research, the identification and comparison of genes within specific pathways across the genomes of various species are invaluable. However, annotating the entire genome is resource intensive, and sequence similarity searches often yield results that are not actually genes. To address these limitations, we introduce Pathway Gene Search (PaGeSearch), a tool designed to identify genes from predefined lists, especially those in specific pathways, within genomes. The tool uses an initial sequence similarity search to identify relevant genomic regions, followed by targeted gene prediction and neural network–based result filtering. PaGeSearch suggests the regions that are most likely the orthologs of the genes in the query and is designed to be applicable for species within five classes: mammals, fish, birds, eudicotyledons, and Liliopsida. Compared with GeMoMa and miniprot, PaGeSearch generally outperforms in terms of sensitivity and positive predictive value, as well as negative predictive value. Also, the exon coverage of gene models from PaGeSearch is higher compared with those in GeMoMa and miniprot. Although its performance shows increased variability when applied to actual biological pathways, it nonetheless maintains an acceptable level of accuracy. Evaluating PaGeSearch across different assembly levels, chromosome, scaffold, and contig shows minimal variation in outcomes, indicating that PaGeSearch is resilient to variations in assembly quality.

Advances in high-throughput sequencing have led to the production of a vast number of eukaryotic genome sequences. These genome sequences and assemblies can be easily accessed and downloaded from various databases, such as NCBI or Ensembl. However, the extent of postassembly processing varies across genomes. For some species, chromosome-level assemblies and annotation information are readily available, whereas for others, only contig-level assemblies exist, and annotation remains incomplete.

Genome annotation, the process of identifying genes and other functional elements within a genome sequence, is a crucial step in analyzing and comparing genomes. To study the genes present within a species’ genome or to compare gene sequences between species, it is essential to identify and describe the genes.

Several genome annotation tools have been developed to date, such as GeneMark-EP+ (Brůna et al. 2020), MAKER (Cantarel et al. 2008), and BRAKER (Hoff et al. 2019), and have significantly enhanced the efficiency and accuracy of genome analysis. Despite these advancements, the substantial amount of data involved, including genome sequences, expressed sequence tags (ESTs), and protein databases, still demands considerable computational resources and time.

In some cases, researchers may be interested in a specific group of genes, such as those in a particular pathway, from one or multiple species to study their conservation, evolution, or functional significance. In these situations, performing whole-genome annotation of various species can be computationally inefficient and time-consuming. To address this problem, sequence alignment (or searching) tools like BLAST (McGinnis and Madden 2004), HMMER (Eddy 1998), and Exonerate (Slater and Birney 2005) are often used. However, these tools have limitations, as they can only find similar parts within gene sequences and often fail to identify the complete sequence of the gene; moreover, they cannot confirm whether the identified regions are actually part of the gene in question.

To tackle these issues, alternative tools like genBlastA (She et al. 2009) and genBlastG (She et al. 2011) were developed to enhance the identification of homologous genes from BLAST search results. genBlastA uses a graph-based algorithm to filter high-scoring pairs (HSPs) into well-defined groups, each of which represents a candidate gene in the target genome. It also introduces a novel edge length metric for identifying gene boundaries. genBlastG takes the groups of HSPs identified by genBlastA and determines gene models by maximizing the similarity to their corresponding queries while examining alignments and neighboring genomic regions for start/stop codons and splicing signals. However, genBlastA and genBlastG rely on BLAST, which can be time-consuming compared with more recently developed and efficient alternatives, such as MMseqs2 (Steinegger and Söding 2017) or DIAMOND (Buchfink et al. 2015). Gene Model Mapper (GeMoMa) also offers an option for predicting a list of reference transcripts in newly sequenced genomes (Keilwagen et al. 2019). GeMoMa is a gene prediction tool that leverages knowledge from well-annotated reference genomes and features an option called “selected,” which filters the transcripts in a given list and annotates only relevant sections of the genome. Also, a recently developed aligner, miniport, that was designed for mapping protein sequences to complete genomes, integrating modern techniques like *k*-mer sketch and vectorized dynamic programming, can be used to find particular genes in the genome (Li 2023).

Biological pathways are networks of interconnected genes and molecules that coordinate and regulate various biological processes within cells, tissues, and organisms. By identifying the genes present in a pathway, researchers can gain insights into their collective functions and how they interact with each other to carry out specific biological processes. This knowledge can help elucidate the underlying molecular mechanisms and regulatory networks that govern cellular processes, providing a deeper understanding of biological systems.

Here, we present Pathway Gene Search (PaGeSearch), a tool that is designed to effectively identify a list of genes within a genome, with a focus on genes associated with specific pathways. Using an initial sequence similarity search to identify candidate regions and subsequently performing gene prediction within these regions, PaGeSearch significantly narrows down the search space. This targeted approach enables more efficient calculations and mitigates computational burden by only focusing on relevant genomic subsets as opposed to the entire sequence. Following this, PaGeSearch uses a neural network model to provide candidates that are the most likely orthologs of the query genes. Only the assembly and query gene sequences are required to run PaGeSearch. The tool also includes a pipeline for downloading orthologous gene sequences for the pathway of interest from the Ensembl database, which can be used as the searching query. PaGeSearch is designed to be applicable across five taxonomic classes—mammals, fish, birds, eudicotyledons, and Liliopsida—and is calibrated based on the genome and gene models of well-annotated model organisms within each class.

We investigated the performance of PaGeSearch on both the genomes of archetype species and those of related species. Furthermore, we compared the tool's performance to that of other competing software, GeMoMa and miniprot. Finally, we applied PaGeSearch to real-world pathway gene identification problems, showing its particular utility for comparative analyses of genes across different species.

## Results

### Overview of PaGeSearch

**Figure 1.**
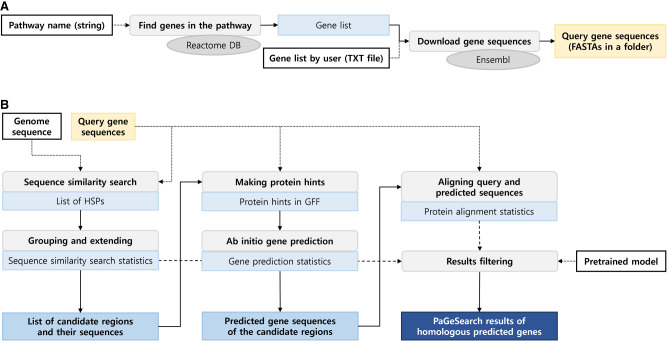
Process of PaGeSearch. (*A*) Genes in the specified pathway are identified from the Reactome database, and their sequences are downloaded from the Ensembl database for use as the PaGeSearch query. (*B*) PaGeSearch initiates by finding seed regions through a sequence similarity search, followed by gene prediction within these regions. The final results are filtered using a neural network model that evaluates sequence similarity, gene prediction metrics, and protein alignment statistics.

The pipeline of PaGeSearch consists of three major parts as illustrated in Figure 1: (1) defining candidate regions, (2) predicting genes at the candidate regions, and (3) validating and filtering the gene prediction results. There are two steps for defining candidate regions: (i) MMseqs2 searching in TBLASTN mode (Steinegger and Söding 2017) and (ii) grouping and extending the MMseqs2 search results. Gene prediction at the candidate regions is achieved in two steps: (i) making protein hints from the query protein sequences using Exonerate (Slater and Birney 2005) and (ii) predicting homology-guided genes using AUGUSTUS (Stanke et al. 2006). To validate and filter the gene prediction results, we used two steps: (i) aligning the query sequences to the gene-predicted sequences with Exonerate and (ii) filtering the results based on scores calculated from protein alignment and gene prediction metrics using neural network models pretrained for each class (mammals, fish, birds, eudicotyledons, and Liliopsida). MMseqs2, Exonerate, and AUGUSTUS were used as third-party tools of PaGeSearch.

### Benchmark

**Figure 2.**
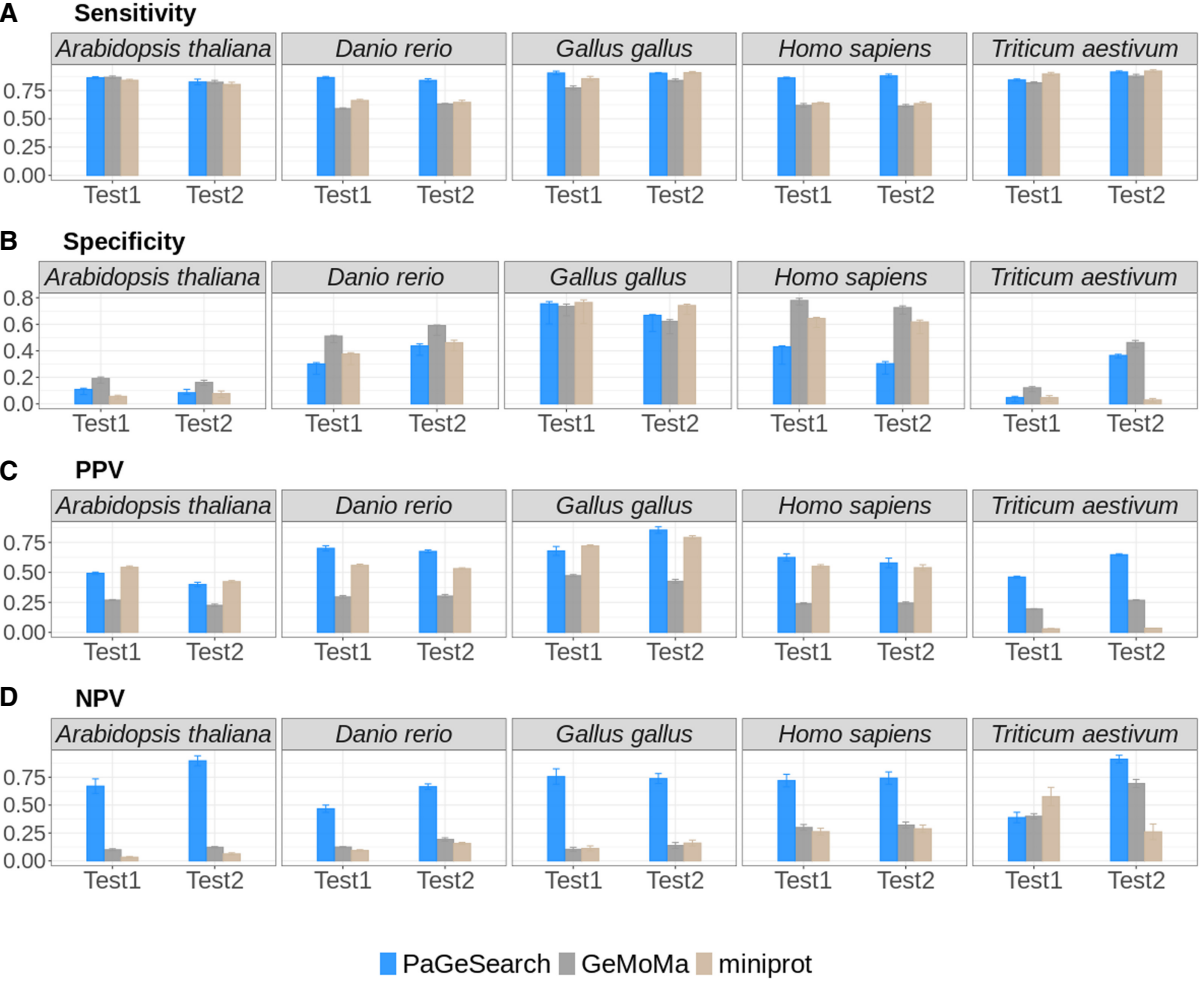
The sensitivity (*A*), specificity (*B*), PPV (*C*), and NPV (*D*) of PaGeSearch, GeMoMa, and miniprot tested on randomly selected genes. The evaluations were conducted on two species that were not used for PaGeSearch results filter model training.

We evaluated the performances of PaGeSearch and the comparable existing tools GeMoMa and miniprot (Fig. 2). GeMoMa can predict genes corresponding to a given list of transcripts, from reference genomes and GFFs (Keilwagen et al. 2019). We used GeMoMaPipeline and provided the list of transcript IDs corresponding to the query genes in the “selected” parameter. We used all transcript IDs that match the gene ID from Ensembl as the input. Default values were used for the remaining parameters. miniprot performs the alignment of a protein sequence to a genome, accommodating affine gap penalties, splicing, and frameshifts (Li 2023). We used the merged FASTA file of PageSearch's query sequences as input and used the default options.

We assessed the sensitivity, specificity, and positive predictive value (PPV), and negative predictive value (NPV) of PaGeSearch, GeMoMa, and miniprot in the two species that were not used for PaGeSearch neural network model training (see Table 1, “Used for benchmark”). Note that in the case of *Arabidopsis thaliana*’s Test2 species, *Solanum tuberosum*, the assembly level is at the scaffold level, and for *Triticum aestivum*’s Test1 species, *Avena sativa*, it is an allopolyploid.

**Table 1.** Species used for training the result filtering model and for testing performance

Used for model construction				Used for benchmark	
Archetype species	Species within order	Species within class	Species within phylum	Test1	Test2
*Homo sapiens*	*Macaca mulatta* (28.8 MYA)	*Ornithorhynchus anatinus* (180 MYA)	*Danio rerio* (429 MYA)	*Mus musculus* (87 MYA)	*Bos taurus* (94 MYA)
*Danio rerio*	*Cyprinus carpio* (90 MYA)	*Lepisosteus oculatus* (321 MYA)	*Homo sapiens* (429 MYA)	*Astyanax mexicanus* (142 MYA)	*Oryzias latipes* (224 MYA)
*Gallus gallus*	*Meleagris gallopavo* (33 MYA)	*Taeniopygia guttata* (91 MYA)	*Homo sapiens* (319 MYA)	*Anas platyrhynchos platyrhynchos* (77 MYA)	*Parus major* (91 MYA)
*Arabidopsis thaliana*	*Brassica oleracea* (26.0 MYA)	*Solanum lycopersicum* (118 MYA)	*Triticum aestivum* (160 MYA)	*Phaseolus vulgaris* (108 MYA)	*Solanum tuberosum* (118 MYA)
*Triticum aestivum*	*Hordeum vulgare* (13.6 MYA)	*Zea mays* (49 MYA)	*Arabidopsis thaliana* (160 MYA)	*Avena sativa* (26.5 MYA)	*Oryza sativa* (47 MYA)

The numbers inside the parentheses indicate the evolutionary distances (millions of years ago [MYA]) between each species and the corresponding archetype species.

Here, sensitivity refers to the proportion of query genes present in the genome that are successfully identified by PaGeSearch, GeMoMa or miniprot in the results. On the other hand, specificity represents the proportion of query genes not present in the genome that are correctly not found by PaGeSearch, GeMoMa or miniprot. PPV is the proportion of correctly identified genes among those found in the results by PaGeSearch, GeMoMa, or miniprot, whereas NPV is the proportion of genes not found in the results by PaGeSearch, GeMoMa or miniprot that are actually not present in the genome. A gene was defined as the genomic region from the start codon and the stop codon.

In evaluating sensitivity, PaGeSearch surpassed both GeMoMa and miniprot in tests involving *Homo sapiens* and *Danio rerio*. For *Gallus gallus*, PaGeSearch showed higher sensitivity in the Test1 species, closely paralleling miniprot with a negligible difference of less than 0.05. In the case of *A. thaliana*, the sensitivities of PaGeSearch and GeMoMa were similar, differing by less than 0.005, whereas miniprot was lower. In tests on *T. aestivum*, miniprot showed the highest sensitivity, followed closely by PaGeSearch and then GeMoMa, with the sensitivity gap between PaGeSearch and miniprot being under 0.02. The average sensitivity across all tested species stood at 0.87, affirming PaGeSearch's proficiency in identifying ∼90% of genes in a specified pathway or list.

Regarding specificity, the ranking in *A. thaliana* and *T. aestivum* was GeMoMa, PaGeSearch, and miniprot, respectively, whereas in *G. gallus*, it was miniprot, PaGeSearch, and GeMoMa. In *H. sapiens* and *D. rerio*, PaGeSearch showed the lowest specificity. A diminished specificity indicates a higher likelihood of false positives, with genes absent in the actual genome erroneously marked as present in the prediction outcomes. In *H. sapiens*, the notable sensitivity advantage of PaGeSearch suggests a possible trade-off scenario.

PaGeSearch consistently achieved a higher PPV compared with that of GeMoMa and miniprot, except in the Test1 species of *G. gallus* and both species in *A. thaliana*. Instances in which PaGeSearch's PPV trailed behind that of miniprot showed a marginal difference not exceeding 0.05. This trend implies that PaGeSearch is less prone to false-positive predictions, likely owing to its refined method of determining the most probable ortholog for each query gene.

The NPV was generally highest for PaGeSearch, except in the Test1 species of *T. aestivum*. A high NPV suggests that genes not identified by PaGeSearch are likely to genuinely lack orthologs.

**Figure 3.**
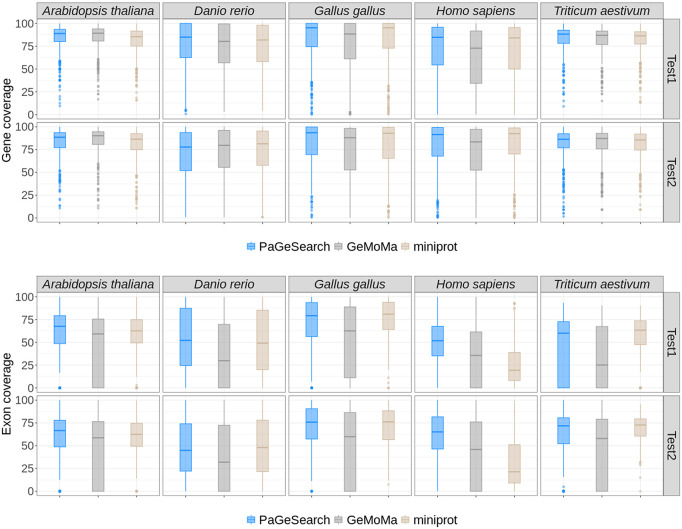
The distributions of gene and exon coverages for true-positive regions, as identified by PaGeSearch, GeMoMa, and miniprot, categorized by archetype species and the test species used for validation.

We also evaluated the extent to which predicted genes and exons covered regions of the actual genes and exons (Fig. 3). The gene coverage was calculated as proportion of the Ensembl gene annotation covered by the predicted gene, and was obtained from only true-positive predictions. The region from the start codon and the stop codon was evaluated as the gene region. Also, we calculated the percentage of coding sequence (CDS) regions of the annotation covered by the predicted exon regions. The overall mean of gene coverage was 0.79 for PaGeSearch, 0.76 for GeMoMa, and 0.78 for miniprot. In more detail, the average gene coverage for PaGeSearch's archetype species ranged from 0.74 to 0.84, and except for *A. thaliana*, it was higher than that of GeMoMa and miniprot.

The average exon coverage was 0.57 for PaGeSearch, 0.42 for GeMoMa, and 0.52 for miniprot. These results indicate that the majority of the gene and exon regions is covered by the predicted regions, and PaGeSearch generally provided higher coverage of the actual gene and exon regions compared with GeMoMa and miniprot.

**Figure 4.**
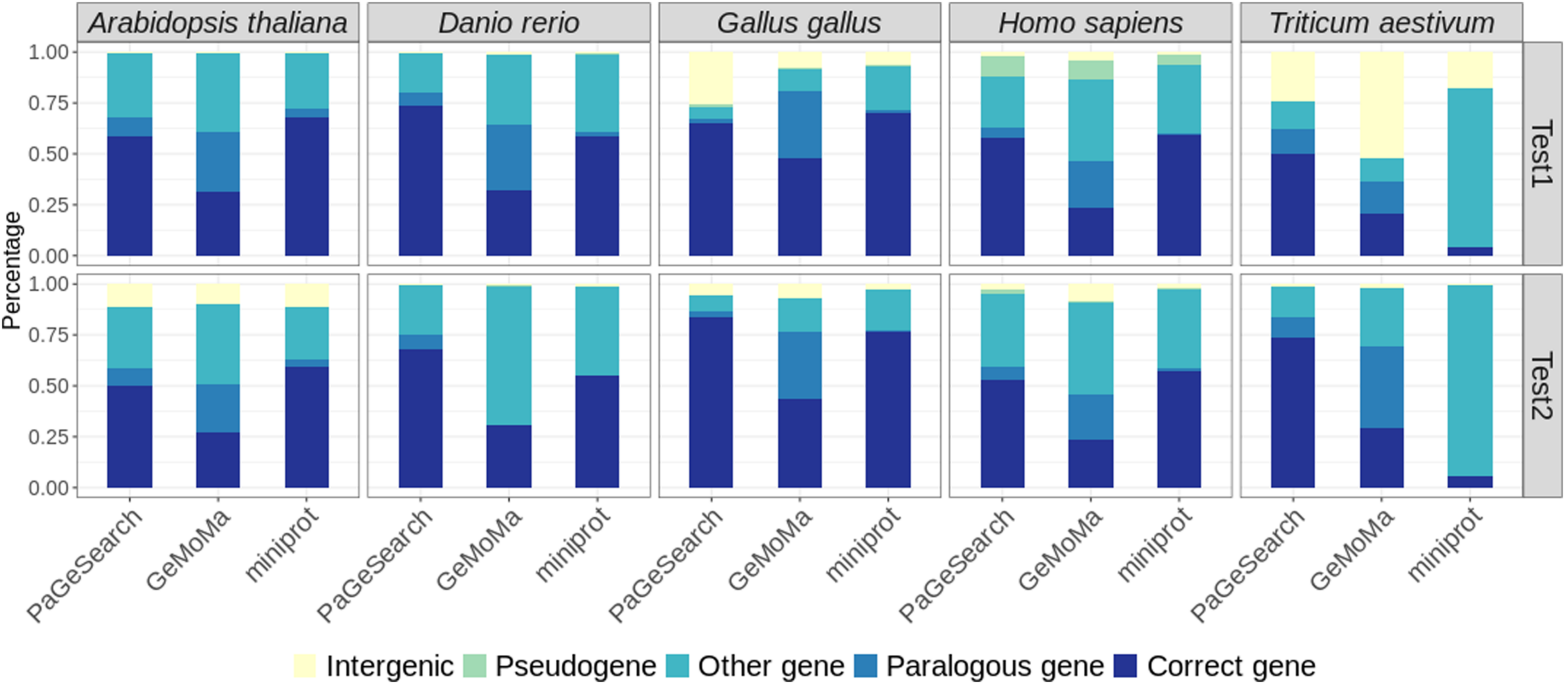
The percentage of annotations of regions identified in PaGeSearch, GeMoMa, and miniprot categorized by archetype species and the species used for validation. “Correct genes” are regions that overlap with the regions of the query genes; “paralogous genes” are regions that overlap with paralogs of the query genes; “other genes” are regions that overlap with protein-coding genes that are neither correct genes nor paralogs of the query genes; “pseudogenes” are regions that overlap with pseudogenes; and “intergenic” are regions that do not overlap with any genes.

Additionally, we annotated the regions identified by PaGeSearch, GeMoMa, and miniprot (Fig. 4). The annotations encompass various gene types: “Correct genes” represent regions that coincide with the query gene regions; “paralogous genes” correspond to regions that intersect with paralogs of the query genes; “other genes” denote regions overlapping with protein-coding genes that are not included in the query, nor are the regions paralogous to it; “pseudogenes” signify regions that overlap with pseudogene annotations; and, finally, “intergenic” refers to regions that do not show overlaps with any gene annotations. To be specific, paralog genes were defined as the orthologs in the test species of genes in the archetype species that are paralogs to the query gene. Pseudogenes refer to those annotated in the GFF as pseudogenes that are not protein-coding genes.

Overall, the occurrence of “paralogous genes” and “other genes” played a substantial role in generating false positives. In PaGeSearch, GeMoMa, and miniprot, the pattern of annotations across different species showed a certain degree of similarity. Generally, GeMoMa identified the highest number of paralogous genes, whereas miniprot detected the fewest. In the results yielded by PaGeSearch, ∼7% were located in intergenic regions, indicating that the majority of PaGeSearch's findings indeed correspond to actual genes. It appears that lower annotation quality is associated with an increased detection of pseudogenes and intergenic regions, especially notable in the Test2 species of *A. thaliana* and avian species. This pattern suggests that PaGeSearch's accuracy may be influenced by the quality of genome annotation, with less refined annotations potentially leading to higher instances of incorrectly identified genomic regions.

### Validation in genomes with different assembly levels

To assess the impact of input genome assembly quality on PaGeSearch performance, we used lower-level assemblies of a test species from the benchmark. Each species was evaluated using both scaffold-level and contig-level assemblies. Therefore, we additionally conducted tests using data at the chromosome level. For the species *G. gallus*, chromosome-level assemblies were the only ones available, hindering the evaluation of lower-level assemblies. The assembly statistics for the used genomes are presented in Table 2.

**Table 2.** The assembly statistics of the genomes used for validation

Archetype species	Species	Assembly level	Contig number	Total length	N50	L50	No. per 100 kb
*Homo sapiens*	*Mus musculus*	Chromosome	61	2,728,222,451	130,530,862	9	2697.8
*Homo sapiens*	*Mus musculus*	Scaffold	12,661	2,801,301,292	1,291,016	585	9412.4
*Homo sapiens*	*Mus musculus*	Contig	224,709	2,477,635,466	24,821	29,731	0
*Danio rerio*	*Oryzias latipes*	Chromosome	25	734,057,086	31,218,526	11	66.9
*Danio rerio*	*Oryzias latipes*	Scaffold	298	772,952,521	31,949,249	12	11.6
*Danio rerio*	*Oryzias latipes*	Contig	2762	737,644,925	971,613	205	0
*Arabidopsis thaliana*	*Solanum tuberosum*	Chromosome	13	810,654,046	61,165,649	6	15,784.6
*Arabidopsis thaliana*	*Solanum tuberosum*	Scaffold	73,172	882,561,945	39,082	5913	1069.2
*Arabidopsis thaliana*	*Solanum tuberosum*	Contig	642,609	827,722,137	1487	144,345	0
*Triticum aestivum*	*Ozyra sativa*	Chromosome	63	375,049,285	29,958,434	6	31.53
*Triticum aestivum*	*Ozyra sativa*	Scaffold	12	375,082,798	30,085,288	6	2.27
*Triticum aestivum*	*Ozyra sativa*	Contig	304	392,277,955	27,391,607	7	0

**Figure 5.**
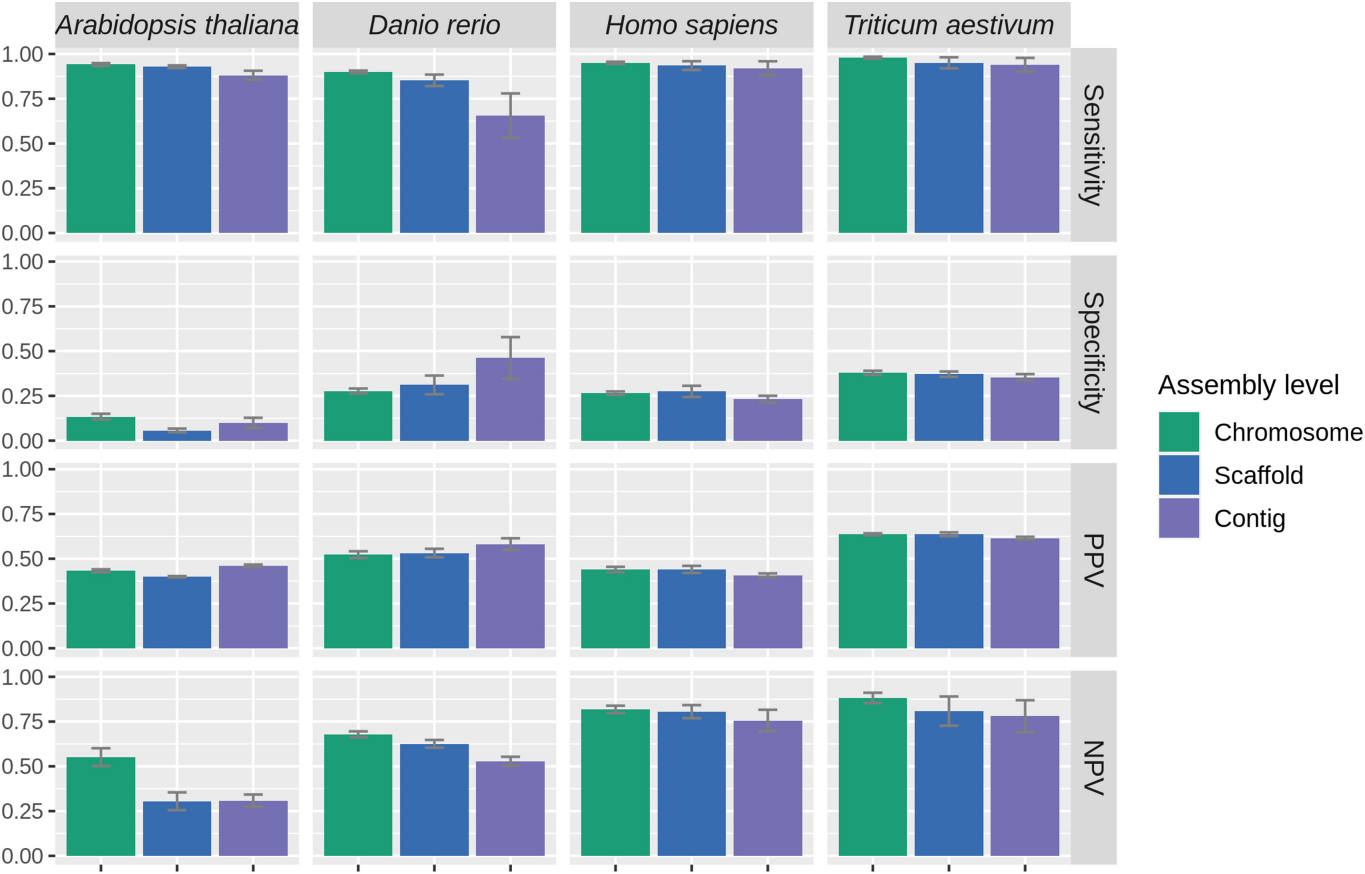
The sensitivity, specificity, PPV, and NPV of PaGeSearch tested on chromosome-level, scaffold-level, and contig-level assemblies.

In these cases, unlike the benchmark in which GFF files were used, sensitivity, specificity, PPV, and NPV were calculated solely based on the presence of gene identification, rather than on the overlap with actual gene regions (Fig. 5).

Lower assembly levels showed a trend toward decreased sensitivity; however, this decrease was not significant. The difference in sensitivity between chromosome-level and scaffold-level assemblies ranged from zero to 0.05 across the four species. For *H. sapiens*, *A. thaliana*, and *T. aestivum* serving as archetypes, the differences between the chromosome and scaffold levels and between the chromosome and contig levels were both within 0.03 and 0.06, respectively. *D. rerio*, however, showed a somewhat larger difference of 0.24 between the contig and chromosome levels.

Specificity and PPV showed no consistent trend across assembly levels. Excluding *A. thaliana* and *D. rerio* as archetypes, the difference in specificity between the chromosome level and other levels remained within 0.04. However, *D. rerio* displayed a specificity difference of 0.18 between the chromosome-level and contig-level assemblies, and *A. thaliana* showed a difference of 0.08 between the chromosome-level and scaffold-level assemblies. PPV remained consistent across the assembly levels. In all cases, the difference between the chromosome-level and either the scaffold-level or contig-level assemblies was within 0.06. NPV generally increased with lower assembly levels, with the exception of *A. thaliana*. The differences between the chromosome-level and lower-level assemblies ranged from 0.02 to 0.15. *A. thaliana* displayed lower NPVs with lower-level assemblies, with differences of 0.25 and 0.24 for the scaffold-level and contig-level assemblies, respectively.

In the benchmark analysis, a gene was only considered a true positive if the detected gene location matched with the reference. However, in this analysis, a gene was considered a true positive simply if it was detected, regardless of its exact location. This approach to gene detection, which focuses solely on the presence or absence of genes without requiring precise location matches, resulted in higher overall sensitivity compared with the benchmark results. Specificity, PPV, and NPV showed variation, being higher in some cases and lower in others compared with the benchmark.

### Application of PaGeSearch to real pathways

**Figure 6.**
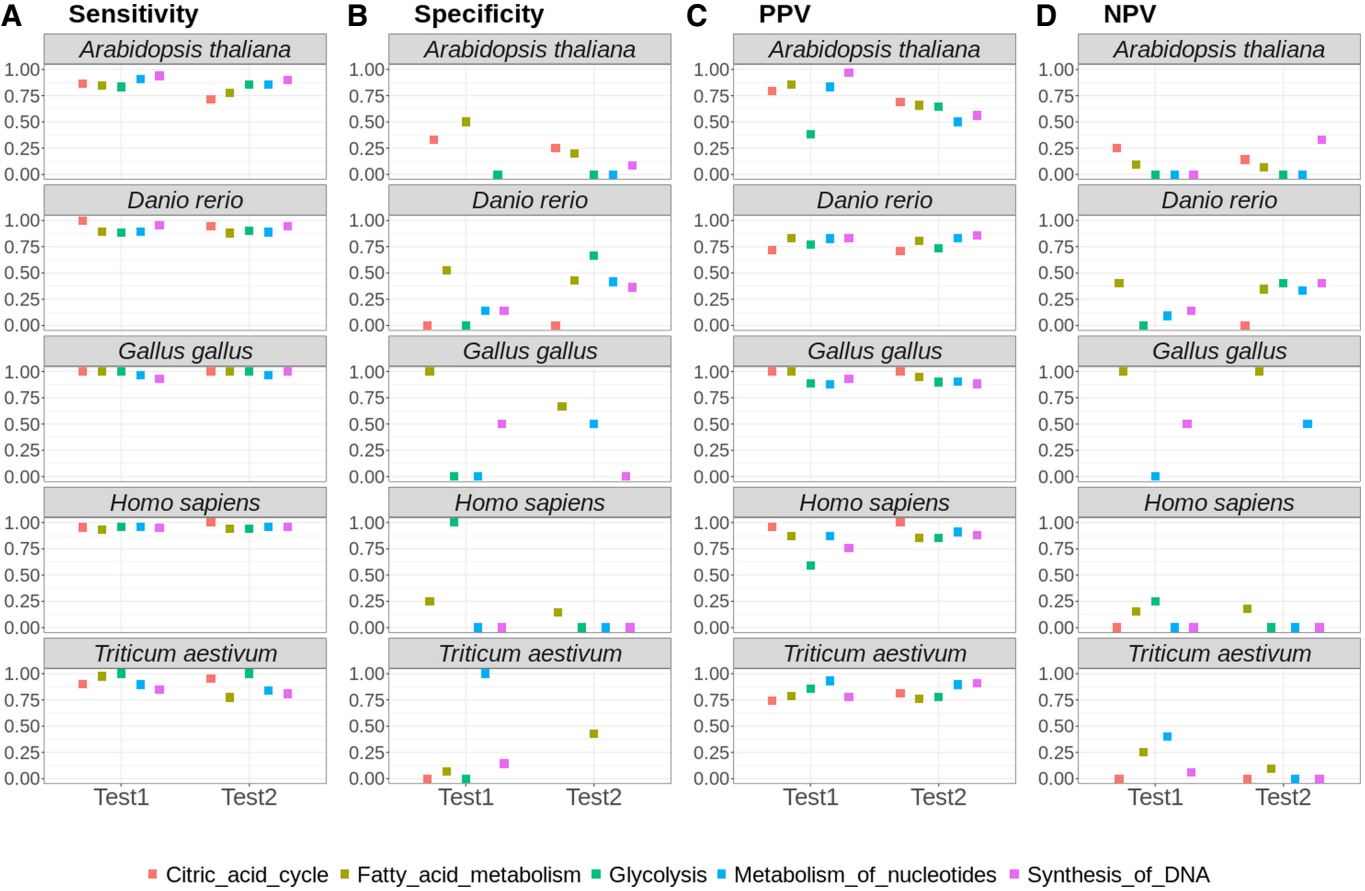
The sensitivity (*A*), specificity (*B*), PPV (*C*), and NPV (*D*) of PaGeSearch tested on five common pathways and two test species.

We selected five common pathways that are shared between animals and plants, and acquired the orthologs of genes included in these pathways using the PaGeSearch gene sequence download pipeline (for description, see Methods, section Data Collection). These orthologs were then used as queries. The search for orthologs was conducted within the largest taxon encompassing the archetype species, excluding the distinct test species. PaGeSearch's performance with actual pathways was assessed in a manner analogous to the benchmark evaluation (Fig. 6).

The average sensitivity across the five pathways for each test species showed a range between 0.82 and 0.99. In most cases, this sensitivity exceeded 0.9. Specifically, for all test species except the two test species of *A. thaliana* and the Test2 species of *T. aestivum*, the average sensitivity consistently remained above 0.9. This trend underscores a generally high level of sensitivity in identifying the correct genes within the specified pathways, indicating the effectiveness of the approach in a majority of the test scenarios.

There were cases in which specificity was unattainable because all of the genes in the pathway had orthologs in the genome being tested. Specificity ranged from zero to one, and the average specificity was 0.25. The PPV for the tests conducted varied, with values ranging from 0.38 to 1.0 across all cases. *A. thaliana* showed the lowest average PPV at 0.70, whereas *G. gallus* showed the highest average PPV at 0.93. Regarding the NPV, there was a tendency for lower- and wider-ranging values when tested on actual pathways compared with random gene sets. The average NPV for each test species spanned from as low as 0.02 to as high as 0.75.

### Runtime and maximum memory usage

**Figure 7.**
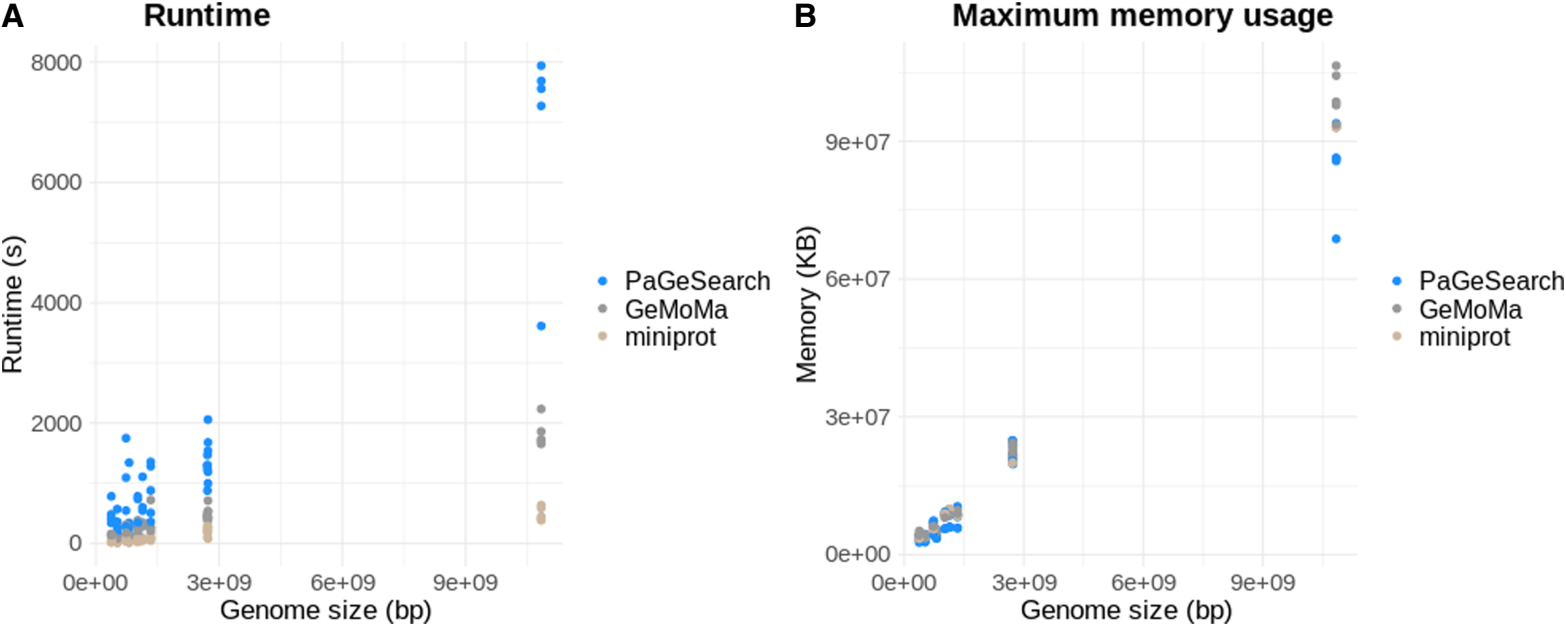
Runtime (*A*) and maximum memory usage (*B*) of PaGeSearch, GeMoMa, and miniprot by genome size. The unit for runtime is seconds, and the unit for genome size is base pairs. The variation in runtime and maximum memory usage observed for the same genome size can be attributed to differences in the number of query genes.

We measured the runtime and maximum memory usage of PaGeSearch, GeMoMa, and miniprot for different pathways and genomes (Fig. 7). Four threads were used for time and memory measures. The runtime increases as the genome size and the number of genes in the pathway increase. For the smallest genome used for validation, *Oryza sativa* (358 Mb), it took 342 to 783 sec to identify 24 to 135 genes in the pathway. For the largest genome, *A. sativa* (10.1 Gb), the runtime was 7941 sec and the maximum memory usage was 89.6 MB in order to find 135 genes in the pathway. In the case of *Mus musculus*, which possesses the largest diploid genome in our study, the runtime for identifying 177 genes was 1677 sec, comfortably under 30 min, and the maximum memory usage was 23.8 MB.

Comparatively, PaGeSearch showed longer runtimes but lower memory usage than both GeMoMa and miniprot. Notably, as the genome size increased, this difference became more pronounced.

## Discussion

PaGeSearch uses a multistep approach, first using similarity searches to pinpoint candidate regions and then carrying out gene prediction within these relatively small genomic fractions. This makes it more efficient than methods that annotate entire genomes. For instance, BRAKER2 processed a 1-Mb nucleotide segment of *A. thaliana*’s genome in 20 to 35 min on 4 CPU (Brůna et al. 2021), whereas PaGeSearch can find 67 genes in the *O. sativa*’s genome, which is larger than the *A. thaliana*’s genome, in 10 min on 4 CPU.

PaGeSearch's longer runtime compared with that of GeMoMa and miniprot appears to be attributable to its gene prediction step, which adds to the processing time. Although PaGeSearch takes longer than GeMoMa and miniprot, it is still significantly quicker than performing a full genome annotation. Furthermore, PaGeSearch tends to have higher sensitivity, PPV, and NPV compared with these tools, and PaGeSearch showed higher exon coverage, presenting more accurate gene models. Therefore, PaGeSearch appears to be a suitable choice for conducting accurate gene searches, providing a balance between efficiency and precision.

The final step of PaGeSearch is filtering hits based on similarity search scores, gene prediction statistics, and alignment scores of the predicted proteins and query proteins. PaGeSearch aims to find the most likely orthologous genes of the genes in the query. This approach enables PaGeSearch to maintain a relatively higher PPV than other tools. Regarding PPV, PaGeSearch generally outperforms GeMoMa and miniprot. PaGeSearch is designed to identify and present the most probable orthologous gene model, and its tendency to yield fewer false positives is expected to facilitate easier interpretation of results by users. This focus on accuracy in predicting the most likely orthologs not only enhances the reliability of the search results but also simplifies the analysis process, enabling users to make more informed decisions based on the provided data with a higher level of confidence in its accuracy.

The performance of PaGeSearch appears to be influenced by the quality of genome annotation. Well-annotated genomes tend to show higher sensitivity and specificity, whereas those with less robust annotations could lead to increased false positives, stemming from unannotated genes, and false negatives owing to incompletely annotated genes. Both sensitivity and NPV are likely affected not only by annotation quality but also by the accuracy of ortholog definitions. Properly defined orthologs between the archetype species and the tested species can significantly impact the occurrence of false positives and negatives. For instance, *S. tuberosum*, the Test2 species of *A. thaliana*, being a scaffold-level assembly, could have contributed to overall lower sensitivity, although the difference may not be substantial.

Validation of PaGeSearch performance across various assembly levels, including the chromosome, scaffold, and contig levels, revealed little overall differences. This suggests that PaGeSearch shows relative robustness to the impact of assembly quality on its results. However, an exception emerged for *Oryzias latipes* contigs, for which sensitivity was notably lower. This can be attributed to the use of assemblies of particularly low quality, because the genome was a draft assembly (Fitzgerald et al. 2022). In fact, the scaffold-level assembly of the same sample yielded a performance comparable to the chromosome-level assembly. *S. tuberosum* showed a decrease in both specificity and NPV with lower assembly levels. This observation could be attributed to the presence of sequences resembling nonexistent genes within improperly assembled regions. In other scenarios, PaGeSearch maintained a certain level of performance. Therefore, analyzing genomes of varying assembly levels with PaGeSearch is unlikely to introduce significant issues provided that the assembly quality is better than draft level. Furthermore, if the assembly is at or above scaffold level, it appears that PaGeSearch can be used without special consideration for assembly quality.

Indeed, although assembly level and quality are not synonymous, it is generally understood that a lower assembly level often implies lower quality. Thus, it is reasonable to consider the assessment of performance across different assembly levels as an indirect evaluation of the impact of assembly quality. Overall, the performance did not significantly differ in lower-level assemblies, suggesting that PaGeSearch can be robustly used regardless of assembly quality to some extent. It should be noted that this comparison was conducted without considering the exact locations of genes, focusing solely on their presence or absence. This approach may contribute to the observed minimal discrepancies. Therefore, when analyzing species that include genomes with lower assembly quality, PaGeSearch appears well suited for determining gene presence or absence rather than for providing precise annotation locations. In other words, when the goal is simply to compare the presence of a specific group of genes across various species’ genomes, PaGeSearch can produce reliable results, even when the assembly quality of the species being compared varies.

**Figure 8.**
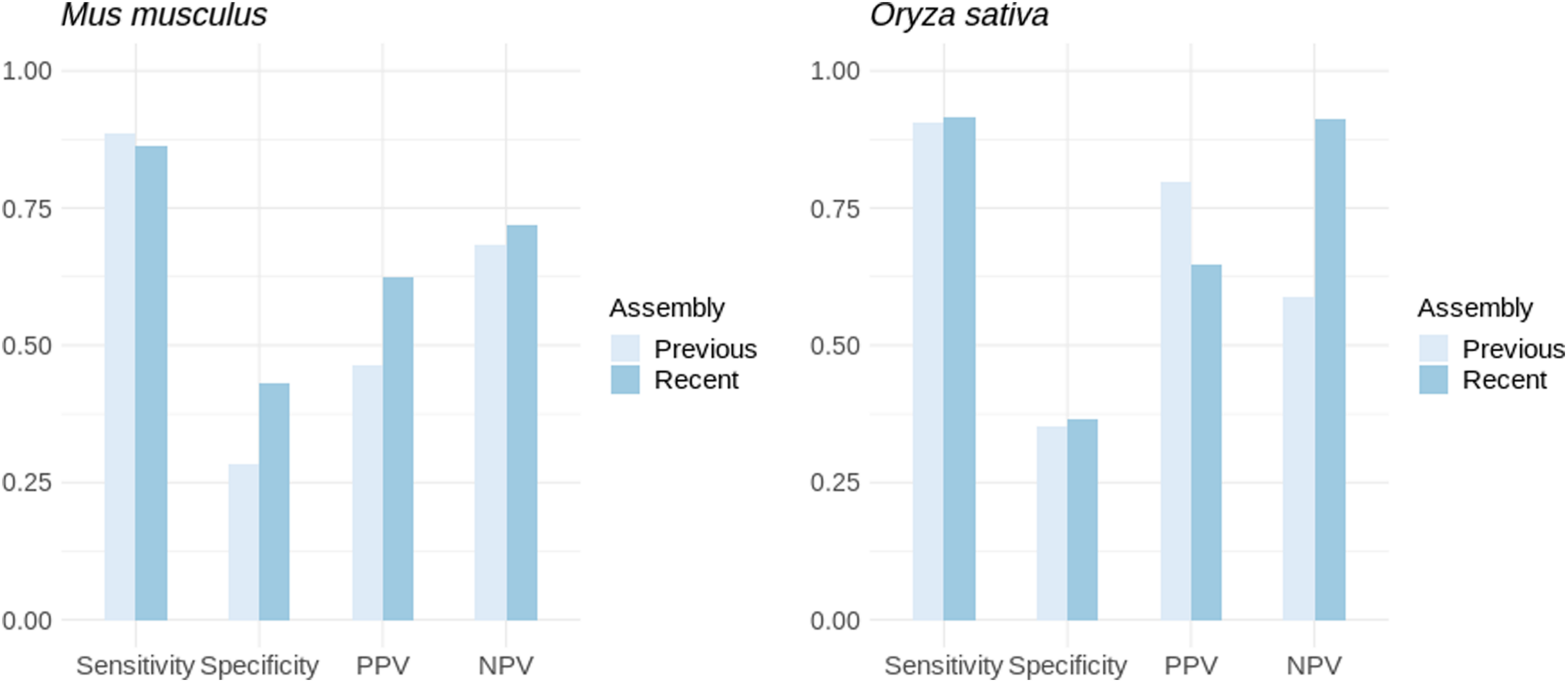
The performance of PaGeSearch tested on the most recent and previous assembly.

To further assess the impact of assembly quality on PaGeSearch's performance, independent of annotation quality, tests were conducted on previous and current versions of the *M. musculus and O. sativa* genomes, for which genome liftOver data were available (Fig. 8). In *M. musculus*, there was no significant difference in sensitivity, although specificity was somewhat reduced. In *O. sativa*, both sensitivity and specificity were largely unaffected by the assembly version. Although both *M. musculus* and *O. sativa* had high-quality previous assemblies, this still indicates a degree of robustness in PaGeSearch to variations in assembly quality.

When dealing with query genes that have a large number of similar genes within the genome, the specificity and PPV of PaGeSearch can be compromised. The low specificity and NPV of PaGeSearch in some species can be attributed to the detection of many similar genes. PaGeSearch operates in two main steps: It first uses a sequence similarity search to identify all possible regions, and then it filters these based on neural network models. Although this approach allows for high sensitivity, it may also result in identifying some false positives, whose sequences are similar to those of the query genes.

To elaborate, when using *A. thaliana* as the archetype, its compact genome and well-annotated status, in which genes with the same function are specifically annotated to have separate names, could lead to fewer true negatives being identified. This affects the performance metrics of both PaGeSearch, GeMoMa, and miniprot manifesting as lower specificity for each tool when working with this species. In contrast, the Test2 species of *T. aestivum*, *A. sativa*, has an alloploid genome, inherently containing more copies of the same genes. PaGeSearch relies on sequence similarity for its search criteria, whereas GeMoMa uses reference sequence and transcript annotation information and miniprot uses protein sequence alignment algorithms. As a result, PaGeSearch is more susceptible to reduced true-negative identification owing to polyploidy, whereas GeMoMa and miniprot are less affected, leading to higher sensitivity in *A. sativa*. Although PaGeSearch's specificity and PPV were influenced by factors such as polyploidy and allopolyploidy, its sensitivity remained consistent.

When validated against actual pathways, the mean sensitivity for all cases was 0.92. This indicates that PaGeSearch can successfully identify >90% of genes within real pathways on average. The overall average specificity across species ranged from 0.04 to 0.43, and the average PPV ranged from 0.61 to 0.94. When applied to common pathways, the sensitivity was higher or comparable to that of the random test set, whereas the specificity and PPV were somewhat lower. Real pathways may contain similar genes within the query. Furthermore, because we tested essential pathways, there may be a number of genes within the pathway query that share high similarity with other genes in the genome. These may have led to the lower specificity, PPV, and NPV in the pathway results compared with the random test set validations, as the existence of genes that have similar sequences with the query genes both within the query and in the genome may yield more false positives. Although the performance shows increased variability when assessed within actual biological pathways, PaGeSearch maintains high sensitivity, and the specificity, PPV, and NPV fall within a range that makes the tool suitable for practical applications in most cases.

PaGeSearch can accurately identify genes within pathways and show its utility across various species within the same classes as the five archetype species. In PaGeSearch, during the gene prediction phase, we explicitly specify the species parameter for AUGUSTUS. The parameter chosen is the archetype species of each taxonomic class, which means that the gene prediction is conducted using parameters from a species different from the one being analyzed. However, because these species belong to the same taxonomic class, they are expected to be evolutionarily similar. Albeit the parameters used were for different species than those being analyzed, this evolutionary closeness lends credibility to the approach. Furthermore, the performance of PaGeSearch tested using both benchmark data and actual pathway data showed consistent accuracy. The results ensure that our methodology is robust, despite the cross-species application of gene prediction parameters. Currently, PaGeSearch can be applied to a class comprising five archetype species. However, we have plans to extend its applicability to a broader range of species, such as insects. This update aims to provide a more comprehensive coverage across diverse taxa. The versatile pipeline offered by PaGeSearch will enable similar analyses to be conducted on species beyond this scope, ensuring its utility for a wide variety of organisms.

In conclusion, PaGeSearch offers a convenient pipeline that can download the sequences of the genes in a pathway and search them in the genome, when only the Pathway ID and genome assembly are provided. PaGeSearch reduces the effort required of searching for gene lists and their corresponding sequences within a pathway, enabling relatively straightforward analysis. Furthermore, the high sensitivity ensures that the majority of genes within the pathway can be identified, whereas PaGeSearch maintains a shorter runtime compared with that of other gene prediction tools. Rather than focusing solely on homologs, PaGeSearch seeks the most probable orthologs, resulting in lower false-positive rates. Because it identifies gene-predicted regions exclusively, its interpretation is simpler compared with similarity search tools such as BLAST. PaGeSearch offers a convenient means for comparative analyses of specific pathways across multiple species, especially when the genome annotation is unavailable or the quality of annotation varies among species.

## Methods

PaGeSearch integrates multiple tools to search regions that are both similar to given gene sequences and are predicted to be genic (Fig. 1). First, seed regions are discovered through a sequence similarity search, followed by the grouping and extension of these regions. Within these candidate regions, homology-guided gene prediction is used to detect genes. Finally, the predicted gene sequence and query gene sequence are aligned, and the results are filtered according to the scores calculated by a neural network model evaluating the similarity search scores and the protein alignment scores.

The models used for result filtering were constructed based on five archetype species and are designed to be generally applicable to other species within the same taxonomic classes: *H. sapiens* (mammals), *D. rerio* (fish), *G. gallus* (birds), *A. thaliana* (eudicotyledons), and *T. aestivum* (Liliopsida). Similarly, other parameters within the pipeline have been adjusted to be applicable to species that fall within the same classes as these five archetype species. The pipeline was implemented using Python programming language, and default parameters were applied for all software components unless otherwise specified. PaGeSearch uses third-party tools for data collection, candidate region identification, protein hint creation, homology-guided gene prediction, and protein sequence alignment.

PaGeSearch also provides a data collection pipeline based on the Reactome pathway database (Gillespie et al. 2022). The pipeline downloads protein sequences of all genes in a given pathway name, as well as the ortholog sequences of those genes for a specific taxon. The sequences are sourced from the Ensembl database (Cunningham et al. 2022) and can be used directly as input for the gene searching pipeline.

### Data collection

As an initial step for pathway gene searching, users can obtain the sequences of genes associated with their pathway of interest. Users have the option to provide either a list of specific genes or Reactome pathway names. When given the pathway name and species names, the set of Ensembl gene IDs related to the pathway is sourced from the Reactome database (Gillespie et al. 2022). Subsequently, gene names and their orthologs are gathered from the Ensembl REST API at http://rest.ensembl.org (Ensembl release 109, February 2023) (Yates et al. 2015). Sequences of orthologs, including their isoforms, are accessed via the Ensembl REST API and are stored in individual FASTA files.

### Candidate region identification through sequence similarity search

First, PaGeSearch consolidates the FASTA files of all genes from the provided list into a unified FASTA file and conducts quality control, purging any sequences with ambiguous amino acids (B, J, O, U, and Z). To boost computational efficiency, only distinct sequences are retained, achieved using SeqKit rmdup (Shen et al. 2016). We use MMseqs2 for the sequence similarity search (Steinegger and Söding 2017). This involves creating a protein database with the query genes and a nucleotide database of the target genome, followed by executing a TBLASTN search.

### Candidate region grouping and extension

MMseqs2 outputs are grouped and expanded for subsequent gene prediction. In particular, HSPs are categorized by gene. HSP regions within a singular gene and strand, which are separated by <100,000 bp, are amalgamated into one extensive region. Each of these regions is then augmented by a margin of 10,000 bp on either side. These enlarged regions are earmarked as potential regions for the ensuing analyses. We generated a summary of sequence similarity search metrics from these candidate regions for future result filtering. Aligned query gene sequences are organized by identical positions and score, retaining only the top five sequences for matches at identical locations for continued analysis. To acquire these sequences, we used SAMtools faidx (Li et al. 2009) and BEDTools getfasta (Quinlan and Hall 2010).

### Making protein hints

For improving gene prediction accuracy, PaGeSearch deploys Exonerate (with the protein2genome model) and a Perl script included in the AUGUSTUS package, exonerate2hints.pl, to make protein hints (Slater and Birney 2005). The source data consist of sequences from the query genes and the candidate regions described in the previous section.

### Homology-guided gene prediction

Homology-guided gene prediction on the candidate regions are performed by AUGUSTUS (Stanke et al. 2006). PaGeSearch searches for complete gene regions, incorporating the protein hints file created in the previous section for the corresponding species model. The protein sequences of the predicted gene regions are extracted using getAnnoFasta.pl, a Perl script from AUGUSTUS. The species parameter for AUGUSTUS was set as the archetype species for each taxonomic class; human (*H. sapiens*) for mammals, zebrafish (*D. rerio*) for fish, chicken (*G. gallus*) for birds, *Arabidopsis* (*A. thaliana*) for eudicotyledons, and wheat (*T. aestivum*) for Liliopsida.

### Protein sequence alignment

PaGeSearch aligns the protein sequences of the query gene sequences to the predicted gene sequences to validate the gene prediction findings. The ungapped model from Exonerate was used to find the best alignment for each predicted gene sequence and obtain the alignment statistics (Slater and Birney 2005).

### Result filtering

PaGeSearch filters the gene prediction outputs by using a pretrained model that integrates metrics from sequence similarity search, gene prediction, and protein alignment. We produced training data sets for each species using PaGeSearch, bypassing the result filtering phase on a selection of thoroughly annotated genomes. We derived all peptide sequences of the archetype species, excluding sequences present in the shortest and longest 10% to omit outliers. Next, we collected genome sequences from four species for each archetype species with varying evolutionary distances, so that we can obtain a comprehensive training data set enabling the general application of the constructed model. The four species were as follows: (1) the archetype species, (2) close species within the same order, (3) species from the same class but a different order, and (4) distant species within the same phylum (Table 1, “Used for model construction”; Supplemental Table S1). The evolutionary distances showcased in Table 1 were extrapolated from the TimeTree database (Hedges et al. 2015).

We labeled the outcomes of gene searching as one if there was an overlap with the query and zero otherwise. To balance the data, synthetic minority oversampling technique (SMOTE) was performed so that the ratio of label one and zero would be one:one using Python library imbalanced-learn (Lemaître et al. 2017). A neural network model was trained using the features in Table 3 and the labels of the outcomes. The neural network model was constructed using the Keras sequential API (https://github.com/fchollet/keras). The model architecture comprised three layers. The initial layer is a dense layer with four neurons, followed by another dense layer with two neurons. The final layer is a dense layer with one neuron, applying a sigmoid activation function, designed for binary classification tasks. To optimize the model, the Adam optimizer was used, with a learning rate of 10^−5^, and the model was compiled with binary cross-entropy as the loss function.

**Table 3.** Features used for result filtering neural network model of PaGeSearch

Step	Feature
Sequence similarity search	Percent identity
Sequence similarity search	Gene coverage
Gene prediction	Percentage of transcript supported by hints
Protein alignment to predicted sequence	Percent identity
Protein alignment to predicted sequence	Percent similarity
Protein alignment to predicted sequence	Gene coverage

The predictions were sorted based on the probability of being labeled as one. When multiple genes were predicted in the same genomic region, only the one with the highest probability was retained. Subsequently, if the same gene was predicted in multiple genomic regions, the prediction with the highest probability was selected. We designed the tool to report the gene model with the highest probability if multiple isoforms of a gene were identified. In cases in which probabilities were equal or very similar (if the prediction probability was higher than 0.95 times the highest prediction probability), multiple predictions were considered. Afterward, only predictions with probabilities greater than or equal to a threshold were selected for further analysis. The threshold was set per archetype species, as the lowest value with a sensitivity of more than 0.95 in the training set. This approach ensures that the most confident and reliable predictions are considered while accounting for cases in which probabilities are close or equal.

### Validation

To assess the performance of PaGeSearch, we conducted evaluations in two species not used for training the result filtering model (Table 1). We used the reference genomes of the species for model construction and performance measure. To evaluate both sensitivity and specificity, we randomly selected 200 genes from the archetype species. The gene set for validation consists of three categories of genes; (1) 100 genes in which orthology is defined between the archetype species and the first test species, (2) 50 genes that are in the archetype species but lack an orthologous gene in the first test species, and (3) 50 genes that are in the archetype species but lack an orthologous gene in the second test species.

If there was overlap between the regions identified by PaGeSearch and the actual query gene regions, these were considered true positives. Regions identified by PaGeSearch that did not overlap with the query were considered false positives. Query regions not present in the PaGeSearch results were classified as false negatives. We considered query genes that were not present in either the searched genome or the PaGeSearch results to be true negatives. The presence of a query gene in a different genome being searched was determined by the existence of orthologous genes. Here, the region of a “gene” was defined as the region between the start codon and the stop codon. This process was repeated five times, and the definition of orthologs was based on the Ensembl annotation and BioMart (Supplemental Table S1).

To further validate PaGeSearch's performance across varying assembly levels, we randomly selected and downloaded a single genome from lower assembly-level category (scaffold and contig) listed in NCBI genome for each species (Kitts et al. 2016). For *S. tuberosum*, despite the reference genome being classified at scaffold level in NCBI, we opted for a data set with a higher scaffold count owing to its assembly appeared as chromosome level in Ensembl (Table 2). The accession numbers for the used genomes are provided in Supplemental Table S2. QUAST was used to generate assembly statistics (Gurevich et al. 2013). We maintained the same gene set used in the previous validation, and positives and negatives were determined based on the presence or absence of gene identification rather than the overlap with actual gene regions.

### Software availability

PaGeSearch source code is available at GitHub (https://github.com/Sohyoung/PaGeSearch) and as Supplemental Code.

## Supplementary Material

Supplement 1

Supplement 2

Supplement 3
